# Magnetic and Magnetoresistive Properties of 3D Interconnected NiCo Nanowire Networks

**DOI:** 10.1186/s11671-016-1679-z

**Published:** 2016-10-19

**Authors:** Tristan da Câmara Santa Clara Gomes, Joaquín De La Torre Medina, Matthieu Lemaitre, Luc Piraux

**Affiliations:** 1 Institute of Condensed Matter and Nanosciences, Université catholique de Louvain, Place Croix du Sud 1, Louvain-la-Neuve, B-1348 Belgium; 2 Instituto de Investigaciones en Materiales - Unidad Morelia, Universidad Nacional Autónoma de México, Antigua Carretera a Pátzcuaro No. 8701 Col. Ex Hacienda de San José de la Huerta, Morelia, 58190 Mexico

**Keywords:** Anisotropic magnetoresistance, 3D nanowire network, Ferromagnetic alloys, Nanochannel-confined electrodeposition

## Abstract

Track-etched polymer membranes with crossed nanochannels have been revealed to be most suitable as templates to produce large surface area and mechanically stable 3D interconnected nanowire (NW) networks by electrodeposition. Geometrically controlled NW superstructures made of NiCo ferromagnetic alloys exhibit appealing magnetoresistive properties. The combination of exact alloy compositions with the spatial arrangement of NWs in the 3D network is decisive to obtain specific magnetic and magneto-transport behavior. A proposed simple model based on topological aspects of the 3D NW networks is used to accurately determine the anisotropic magnetoresistance ratios. Despite of their complex topology, the microstructure of Co-rich NiCo NW networks display mixed fcc-hcp phases with the c-axis of the hcp phase oriented perpendicular to their axis. These interconnected NW networks have high potential as reliable and stable magnetic field sensors.

## Background

The particular architectures and high degree of nanowire (NW) interconnectivity of three-dimensional (3D) NW networks make them attractive nanodevice components for a wide range of applications in energy harvesting/storage systems [1–3], electronic sensing devices and actuators [4–6], catalysts [7], electrochromic elements [8], solar cells [9], biosensors [10], and bio-analytical devices [11, 12]. Magnetic NW networks are also expected to play an important role in the development of next-generation multifunctional devices like 3D superstructures with controlled anisotropy and microwave absorption properties [13] and for the storage and logic operation of information carried and processed by domain walls flowing along them [14]. Template-assisted synthesis has proven to be a versatile bottom-up approach for low-cost, reliable, and large-scale fabrication of 3D NW networks with controlled size, geometry, composition, and surface morphology. Typically, these 3D NW networks are obtained by simple electrochemical deposition within the hierarchical nanopores of a suitable template. Among the various 3D nanoporous templates used for this purpose, track-etched polymeric membranes [7, 13] is the most promising as dense networks of crossed cylindrical nanopores can be obtained through sequential polycarbonate (PC) film irradiation with energetic heavy ions at different incidence angles, followed by selective chemical etching of the ion tracks within the polymer film [15]. This template-assisted synthesis enables excellent control over the geometry, chemical composition, and nanoarchitectures that can be the framework for nanoscale devices and systems. The 3D nanoarchitecture also facilitates the ability to perform electron transport measurements through the interconnected, crossed nanowires (CNWs). In this work, we demonstrate the suitability and reliability of using CNWs of different magnetic materials to obtain tunable magnetoresistive behavior. Interconnected NW networks made of electrodeposited NiCo alloys were chosen for the present study because of the interest in these alloys for a wide variety of applications, including magnetic storage systems [16, 17], magnetic and microresonator sensors [18, 19], fuel cells [20], microelectromechanical systems (MEMS) [21], hydrogen storage [22], and materials as catalysts [23]. It is shown that magnetic alloy CNWs of controlled composition can be easily obtained through careful control of the deposition potential and using different electrolytic solutions. The subtle interplay between magnetic and magnetoresistive properties, and structural features is found to be crucial to tailor the magnetic and transport properties of such CNWs. Finally, in order to precisely determine the anisotropic magnetoresistance ratio of NiCo CNW networks from simple magneto-transport measurements, we propose a model that considers the spatial arrangement of NWs in the 3D network.

## Methods

The 20- *μ*m thick crossed nanoporous templates have been prepared by performing a sequential multi-step exposure of energetic heavy ions, at various angles with respect to the normal of the PC film surface. For the present study, a PC film was subjected to a first irradiation step over a wide angular range from −45° to +45° with respect to the normal axis of the PC surface. Next, for the second irradiation step the film was rotated in the plane by 90° and re-exposed to the same angular variable irradiation flux to form finally a complex 3D nanochannel network. The angular standard deviation in both irradiation steps was ±5° maximum around the target maximum angle. Both irradiation steps with quasi-continuous angular variation are a key requirement to obtain highly interconnected porous networks. The latent tracks were chemically etched in 0.5 M NaOH aqueous solution at 70 °C to form 40-nm diameter nanopores, following a previously reported protocol [15]. The as-prepared polymer membrane containing dense networks of 3D interconnected cylindrical nanopores [7, 13] was designed with volumetric porosity of approximately 20 %. In a second stage, the PC templates were coated on one side using an e-beam evaporator with a metallic Cr/Cu bilayer to serve as cathode during the electrochemical deposition. The thickness of the thin adhesion layer of Cr was 10 nm, while for a uniform and consistent nanopore coverage withstanding the electrodeposition process, the Cu film thickness was set to 150 nm.

**Fig. 1:**
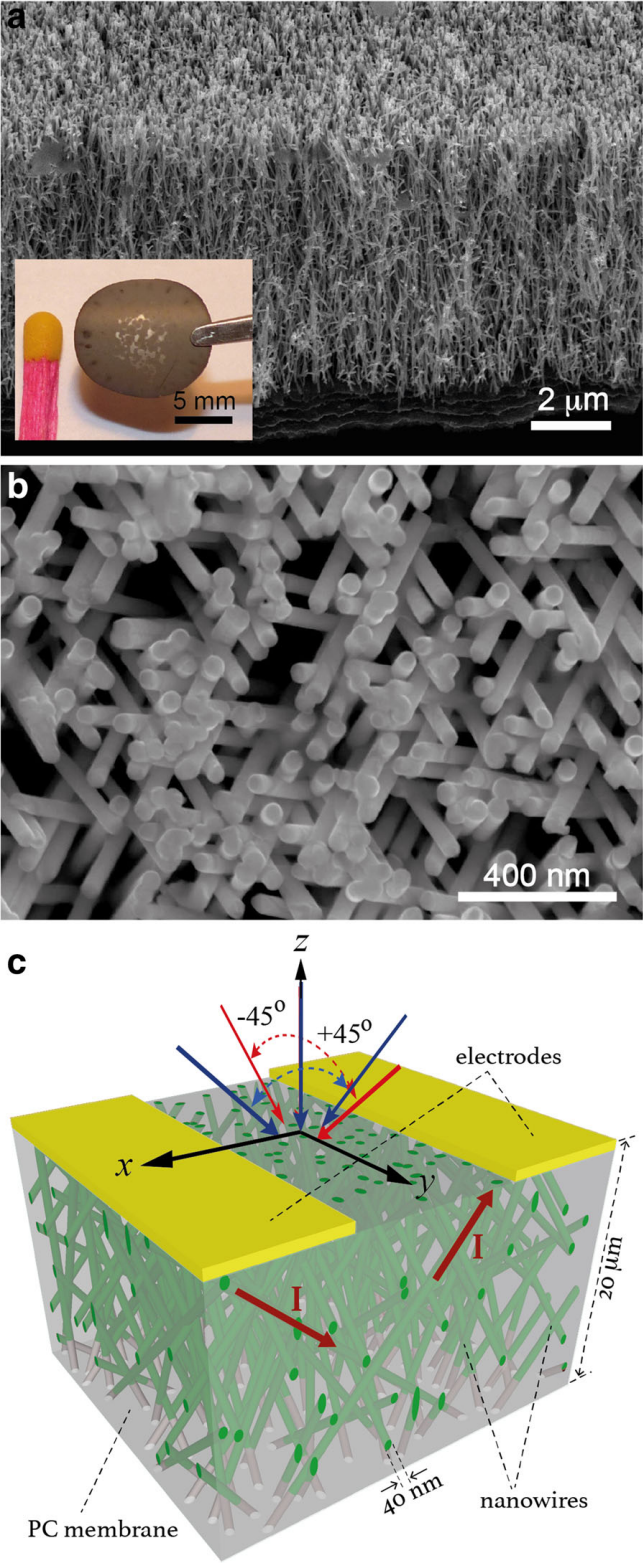
**a** Low-magnification tilted view SEM image of an interconnected network of electrodeposited 3D Ni CNWs, with NW diameter of 40 nm, obtained after complete dissolution of the host PC membrane. The *inset* displays an optical image showing the size and mechanical robustness of the macroscopic self-supporting network. **b** Top view of the dense Ni CNW network of **a**. **c** Schematic representation of a 3D PC membrane featuring cylindrical nanopores with a mean diameter of 40 nm and partially filled with CNWs. An electrode design for two-probe electrical measurement is also shown

Nickel, Cobalt, and Ni _*x*_Co_1−*x*_ (0≤*x*≤1) CNW networks were grown by electrodeposition into interconnected pore PC templates at room temperature in the potentiostatic mode using a Ag/AgCl reference electrode and a Pt counter electrode. Electrodeposition of Ni and Co CNWs was carried out at the respective constant potentials of −1.1 and −0.95 V using the following electrolytes: 262.8 [g l ^−1^] NiSO_4_ + 30 [g l ^−1^] H_3_BO_3_ at pH 3.4; and 238.5 [g l ^−1^] CoSO _4_·7H_2_O + 30 [g l ^−1^] H_3_BO_3_. The as-prepared Co solution has a pH value of 3.6, which was increased up to 5.0 by the addition of NaOH [24]. In the case of the Ni _*x*_Co_1−*x*_ (20 *%*≤*x*≤75 *%*) alloyed CNW networks, electrodeposition was carried out at potentials in the range from −0.85 to −2 V using the electrolyte 604.5 [g l ^−1^] Ni(SO_3_NH_2_) _2_·4H_2_O + 112.5 [g l ^−1^] CoSO _4_·7H_2_O + 30.9 [g l ^−1^] H_3_BO_3_. For alloys with 60 *%*≤*x*≤90 *%* electrodeposition was done for deposition potentials from −1 to −2 V using the same electrolyte but with a Co concentration of 28.1 [g l ^−1^] CoSO _4_·7H_2_O instead. The pH of the as-prepared NiCo solution was lowered down to 2.2 by addition of H_2_SO_4_. The morphology of the nanostructured CNW networks was characterized using a field-emission scanning electron microscope (FE-SEM). For the electron microscopy analysis, it was necessary to remove the PC template. This was done by first etching the cathode using a I2:KI (0.1:0.6 M) solution and then dissolving the polycarbonate with dichloromethane. X-ray diffraction (XRD) measurements have been carried out by using the line *λ*=0.154056 nm of a CuK *α*1 radiation source, coupled with a Siemens D5000 diffractometer. Magnetization curves were obtained at room temperature in the out-of-plane (OOP) and in-plane (IP) directions of the CNW network film, using an alternating gradient field magnetometer (AGFM-Lakeshore) with a maximum applied field of ±10 kOe. The magneto-transport measurements were performed at 20 and 290 K while sweeping a magnetic field between ±10 kOe in the IP and OOP directions. The experimental setup for magneto-transport is based on a four/two-probe measurement system. A mechanical mask was used for selective local removal of Cu cathode by wet chemical etching using the iodine-based solution and for creating an electrode design at the surface of the filled template [Fig. 1 c]. In this configuration, the current is directly injected to the branched CNW structure from unetched sections of the Cu cathode thanks to the high degree of electrical connectivity of the CNWs.

It should be noted that the results obtained by four- and two-probe measurements were the same, as the typical resistance values of the prepared specimens (in the range of few tens of *Ω*) were usually much larger than the ones attributed to the corresponding leads and contacts to the sample. The measured samples were about 1 cm long, and the electrical contacts were directly made by Ag paint. For each sample, the input power was kept below 0.1 *μ*W to avoid self-heating, and the resistance was measured within its ohmic resistance range with a resolution of one part in 10^5^.

## Results

Figure 1 a shows the tilted view SEM micrograph of the self-standing 40-nm diameter Ni CNW network after the complete dissolution of the PC membrane. As seen, the CNWs network exhibits a complex interconnected structure providing a high degree of electrical connectivity and good mechanical stability, as the entire 3D-CNW networks (with an area of ∼1 cm^2^) are self-supported and can be easily handled by tweezers, as seen in the inset to Fig. 1 a. The topological structure of the porous membranes is a key feature in giving rise to robust network architectures made up of self-standing magnetic CNW networks. The top-view SEM micrograph of Fig. 1 b shows a homogeneous orientation of the cylindrical NWs along the angular range of ±45°. This figure also displays a detailed view of the complex structure of the interconnected NWs.

**Fig. 2:**
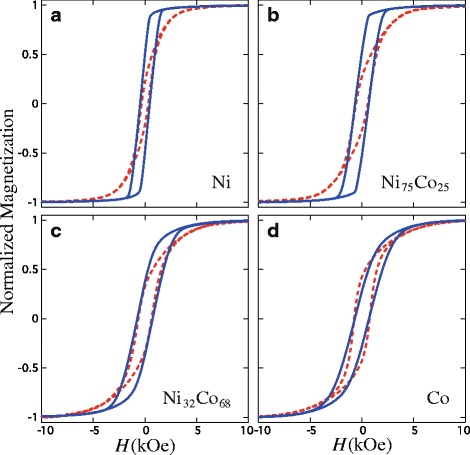
Hysteresis loops measured with the field applied in the OOP (*continuous lines*) and IP (*dashed lines*) directions of the PC membrane in **a** Ni, **b** Ni_75_Co_25_, **c** Ni_32_Co_68_, and **d** Co CNW networks

Figure 2 a–d show room temperature hysteresis loops measured with the field applied in the OOP and IP directions for Ni _*x*_Co_1−*x*_ (0≤*x*≤1) CNWs. As seen from the comparison between the hysteresis loops in the IP and OOP directions shown in Fig. 2 a, the easy axis of magnetization for the Ni CNW network lies in the OOP direction; however, the magnetic behavior of the 3D networks in Fig. 2 b–d become more isotropic as the Co content in the alloy progressively increases to the full Co CNW network. In this case, the very similar shape of both hysteresis loops in Fig. 2 d suggests a nearly magnetically isotropic behavior, which is consistent with the presence of a magnetocrystalline (MC) anisotropy contribution that lies in the direction perpendicular to the long axis of the NWs [24]. Indeed, such a MC contribution directly opposes the shape anisotropy of the NWs, which gives rise to a competition between both anisotropies and then to a decrease of the effective magnetic anisotropy of the CNW network.

**Fig. 3:**
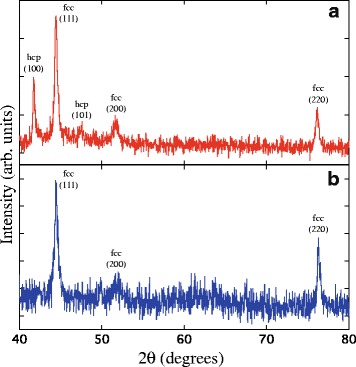
X-ray diffraction patterns of **a** the Ni_32_Co_68_ and **b** Ni_75_Co_25_

The hysteresis loops obtained on the CoNi alloys corroborate the XRD results. Figure 3 a, b present the typical XRD spectra of Co-rich and Ni-rich CNW networks, respectively. For Ni_32_Co_68_ CNWs, two peaks related to the hcp (100) and hcp (101) planes are found (JCPDS 05-0727), as shown in Fig. 3 a, indicating that a mixture of the fcc and hcp phases are present in Co-rich NiCo CNWs, in agreement with the binary phase diagram of Co-Ni alloys showing a mixed hcp and fcc region between about 65 and 100 % Co, as previously observed in electrodeposited thin films [25–27] and nanowires [28, 29]. For Ni_75_Co_25_ CNWs in Fig. 3 b, three peaks corresponding to the (111), (200), and (220) planes of the fcc structure (JCPDS 01-1260 and 15-0806) are observed. Then, in Co-rich NiCo CNW networks the hcp phase begins to appear when its Co content exceeds 65 %. Furthermore, the presence of these hcp peaks implies that for Co-rich NiCo CNWs the hcp c-axis is predominantly oriented perpendicular to the nanowire axis, as for pure Co NWs [24].

**Fig. 4:**
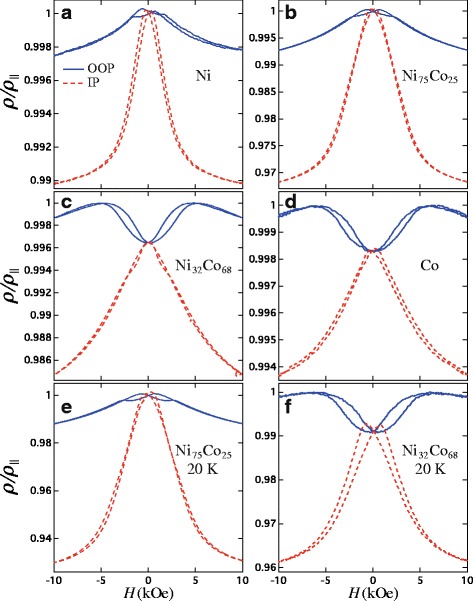
Magnetoresistance curves for Ni _*x*_Co_1−*x*_ (0≤*x*≤1) CNW networks measured **a**–**d** at 290 K and **e**, **f** at 20 K, by applying the external field in the OOP (*continuous lines*) and IP (*dashed lines*) directions

Anisotropic magnetoresistance (AMR) measurements in transition ferromagnetic metals and their alloys are consistent with changes in the resistivity as the angle between the magnetization and current directions is modified. The physical mechanisms giving rise to the AMR effect implies an anisotropy in the transport properties that can primarily be ascribed to the presence of the spin-orbit coupling and the magnetic ordering. In the case of arrays of parallel NWs, the AMR ratio was directly determined from the high and low resistance states (*ρ*_||_ and *ρ*_⊥_) where the magnetization is respectively parallel and perpendicular to the current which flows along a single direction, parallel to the NWs axis [30, 31]. Figure 4 a–d show resistance curves measured at 290 K with the external field in the OOP (continuous lines) and IP (dashed lines) directions for interconnected NiCo CNW networks. As observed, the maximum resistance is reached near zero applied field for both directions as the Ni content in the alloy dominates (see Fig. 4 a, b), which is consistent with remanent magnetization states where the magnetization tends to be aligned along the nanowire axis, due to shape anisotropy as suggested by Fig. 2 a, b. Conversely, the decrease of the resistance near zero field as the Co content in the alloy is dominant (see Fig. 4 c, d) is consistent with the decrease of the remanent magnetization (see Fig. 2 c, d) as a result of the misalignment of the magnetization with respect to the NWs axis, due to the competing MS and MC anisotropies. The same magnetic behavior was found on the Co-rich Ni _*x*_Co_1−*x*_ CNWs with *x*<35 %. Besides, with the exception of the difference in increase between the resistance curves measured at 20 K for both directions, its overall behavior is very similar to the one for the curves measured at 290 K. It was found that the measured electrical resistances were very stable with time, by repeating the experiment several times on the same sample with almost no perceptible changes (of the order of 1 %, which also may be ascribed to the slight variation of resistivity as a function of ambient temperature). Since the interconnected nanowire architecture is mechanically stable after chemical dissolution of the polymer membrane, similar magneto-transport measurements were performed on a selected self-supporting 3D CNW structure, giving rise to the same magnetoresistive response.

## Discussion

In order to quantitatively analyze the magneto-transport properties for the distinct CNW networks shown in Fig. 4, a model has been elaborated to account for each anisotropic magnetoresistance (AMR) contribution due to all the nanowire orientations in the 3D CNW network. According to the AMR relation [32], the electrical resistivity of magnetized materials depends on the relative orientation (*θ*_0_) between the electrical current along the NWs and the magnetization in the applied field direction, that is

1 $$ \rho (\theta_{0})=\rho_{\perp}+(\rho_{||}-\rho_{\perp})\cos^{2}\theta_{0},  $$

where $\frac {\pi }{4}\leq \theta _{0}\leq \frac {\pi }{2}$ for the magnetization lying in the IP direction and *ρ*_||_ (*ρ*_⊥_) is the resistivity of the CNW network in the high (low) resistance state when the local magnetization and current paths are parallel (perpendicular) to each other. By virtue of the uniform distribution of nanowire orientations in the range [−*π*/4,*π*/4] with respect to the normal plane of the membrane, resistance measurements correspond to average magnetoresistive values resulting from the contributions of all the current paths with different orientations with respect to the applied field direction. The resistance value at saturation ($\bar {\rho }$) in the IP configuration can be obtained by averaging Eq. (1) over the different equivalent positive and negative orientations, that is, over the range $\frac {\pi }{4}\leq \theta _{0}\leq \frac {\pi }{2}$. This gives

2 $$\begin{array}{@{}rcl@{}} \bar{\rho}&=&\frac{1}{L}\int_{\pi/4}^{\pi/2}\rho (\theta_{0})d\theta_{0} \\ &=& k\rho_{||}+\left(1-k\right)\rho_{\bot}, \end{array} $$

where *L*=*π*/4 is the interval length of angles and $k=\frac {1}{2} - \frac {1}{\pi }$. Assuming that *ρ*_||_ and $\bar {\rho }$ are known, the resistance state (*ρ*_⊥_) for the perpendicular configuration between $\vec {M}$ and $\vec {I}$ can be determined from Eq. (2), that is

3 $$ \rho_{\perp}=\frac{\bar{\rho}-k\rho_{||}}{1-k}.   $$

Using Eq. (3), the AMR ratio is finaly obtained as

4 $$ \frac{\Delta\rho}{\rho}=\frac{\rho_{||}-\rho_{\perp}}{\rho_{av}},   $$

where *ρ*_*av*_=(1/3)*ρ*_||_+(2/3)*ρ*_⊥_ is the average magnetoresistance in 3D systems.

**Fig. 5:**
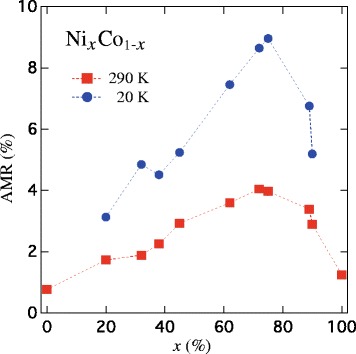
Variation of the AMR ratio vs. the Ni content at 20 and 290 K for Ni _*x*_Co_1−*x*_ alloyed CNW networks

Figure 5 shows the as-determined AMR ratio vs. Ni content (*x*) from the measured IP resistance at saturation ($\bar {\rho }$) as input parameter into Eqs. (3) and (4), at 20 and 290 K for the different Ni _*x*_Co_1−*x*_ CNW networks. The observed variation of the AMR ratio as a function of *x* is consistent with previous reports on electrodeposited films and metallurgically processed NiCo alloys [26, 27, 32], where the maximum at *x*≈75 % for both temperature values is attributed to a magnetostriction and MC constants close to zero and to a saturation magnetization of about one Bohr magneton per atom for that particular alloy composition [32]. As seen in Fig. 5, maximum AMR ratios of about 4 % are obtained at room temperature on the Ni_75_Co_25_ CNWs sample, which is slightly smaller than the one reported in films and bulk alloy with the same composition, showing a maximum AMR ratio of ≈6 %. However, the enhancement of the AMR ratio at low temperature is much reduced compared to bulk alloys. For example, for Ni_75_Co_25_ bulk material, one has *Δ* *ρ*/*ρ*=20 % at *T*=20 K but only 9 % for CNWs of the same composition. This is due to the larger residual resistivity *ρ*_0_ due to scattering by static lattice defects and surface roughness in CNW structures. Indeed, the residual resistivity ratio (*ρ*_290*K*_/*ρ*_20*K*_) for the Ni_75_Co_25_ CNWs sample was only 2.45. Using Matthiessen’s rule, *ρ*(*T*)=*ρ*_0_+*ρ*_e−ph_(*T*) with *ρ*_e−ph_ the temperature-dependent electron-phonon contribution to the resistivity value of approximately 8 *μ* *Ω*cm at room temperature for this alloy composition [26], we estimated *ρ*_0_ to be around 5.5 *μ* *Ω*cm which is about two times greater than the values for films and bulk alloy with the same composition [26, 27, 32]. Given the AMR ratio (4 %) for this particular nanowire alloy, the change in resistivity between the low and high resistance state is about 0.5 *μ* *Ω*cm at room temperature. The enhancement of the AMR ratio at low temperature can be attributed to the smaller resistivity of the alloy nanowire network.

## Conclusions

In this work, we demonstrate the ability to perform reliable magneto-transport measurements through CNW networks fabricated from pure magnetic metals and alloys by electrodeposition into the crossed and interconnected nanochannels of track-etched polymer membranes. The magnetic properties and magnetoresistive response as a function of the angle between the magnetic field and the plane of the nanowire network films made of NiCo alloys can be controlled by a suitable choice of the composition of the ferromagnetic alloy. Both structural and magnetic characterization reveal the presence of mixed fcc-hcp phases as the Co content increases, with the c-axis of the hcp phase oriented perpendicular to the nanowire axis. The AMR values were accurately determined using a model that considers the spatial arrangement of NWs in the 3D network. The measured electrical resistances were very stable with time, so these CNW structures can be used to obtain reliable and stable magnetic field sensors. Finally, the present work opens up the possibility for a controlled template-assisted synthesis of complex nanowire-based architectures with excellent control over geometrical features and chemical composition, leading to tunable magnetic and magneto-transport properties.
